# The Neuron and Disease

**Published:** 1897-10-30

**Authors:** William R. Gowers

**Affiliations:** Physician to the Hospital, Consulting Physician to University College Hospital


### MEDICAL PROGRESS AND HOSPITAL CLINICS.

[*The Editor will be glad to receive offers of co-operation and contributions from members of the profession. All letters should be addressed to* THE EDITOR, AT THE OFFICE, 28 & 29, SOUTHAMPTON STREET, STRAND, LONDON, W.C.]

### THE NEURON AND DISEASE.

Abstract of Introductory Address delivered at the National Hospital for the Paralysed and Epileptic, October 5th, 1897, by Sir WILLIAM R. GOWERS, M.D., F.R.S., Physician to the Hospital, Consulting Physician to University College Hospital.

During the last few years a revolutionary change in our conceptions as to the structure and functions of the nervous system has been brought about by the method of successive staining with chromium and silver introduced by Golgi. This reveals in hitherto unknown distinctness the course and form of the processes of the nerve-cells in the grey matter. It has shown that these processes, till now believed to join, are in truth discontinuous, ending freely in the ground substance or matrix in which the cells lie, and in which only the dimmest trace of structure can be seen. The cells and their processes thus form a series of discontinuous elements, and it is to these elements that the name of "neurons" was first applied by Waldeyer eight years ago. What was formerly called the axis-cylinder process is now known as the "axon," while the remaining, branching, processes are termed "dendrons." The attempt to replace this now generally adopted nomenclature by one more literally correct is confusing, and to be deprecated.

The first important new fact, then, is the discontinuity of the neurons; the second is that, as pointed out by Max Schultze 30 years ago, the axons are not single fibres, but are made up of a number of parallel fibrils, perhaps as many as 50 in each. These are composed of a substance called hyaloplasm, and are separated from one another by a similar but slightly different material termed spongioplasm. It is significant that these bodies, the distinction between which is ordinarily well-nigh imperceptible, should respond so differently and so characteristically to the chemical substances contained in the new staining reagents. This fact throws much light on the selective power of bodies, such as strychnine, atropine, lead, and arsenic, for various particular elements of the nervous system. The new facts at once clear up another hitherto insoluble mystery, the division of axis-cylinders near their terminations. This is now seen to be merely the separation for distributive purposes of the fibrils whereof they are composed. Furthermore, Max Schultze observed and figured—though he did not describe it in so many words—the uninterrupted passage of these fibrils through the nerve-cells. Hence we arrive at a new conception of the functions of the latter, which are no longer the manufactories of nervous impulses, but thereby the nutritive centres of the nerve-fibrils. From them a thin line of protoplasm—thickening at intervals to enclose nuclei—spreads along each axon to subserve its nutrition. Propulses run to the cell along the dendrons, and from it along the axons, and the conditions of their generation and transmission in the centres and at the periphery are thus harmonised. The conception of the cell as the originator of nervous energy thus becomes not only unnecessary, but even incongruous. The exact nature of the process which goes on in the matrix between the extremities of the dendrons and axons is still uncertain. In a peculiar colourless shrimp these extremities have, it is said, been seen to move about among one another like cilia, but it is doubtful whether our present powers of investigation would permit of the accurate observation of such a phenomenon.

The difficulty of conceiving the origin of co-ordinated impulses from the terminations of neurons in the matrix is lessened by the consideration that all matter is built up of a similar multiplicity of minute elements; the same holds with physical phenomena, such as light.

Turning to the application of these facts to nervous pathology we find in them at once an explanation of secondary degenerations in the grey matter. The mystery of the cessation of degeneration of the motor tracts in the cord at the motor nerve-cell is cleared up at once when we discover that the supposed continuity between the two does not exist. The axons of the pyramidal tracts degenerate, but the dendrons of the motor cells in the anterior cornua escape. Not less striking is the elucidation of the action of toxic substances on the nervous elements. The poison of tabes—produced, no doubt, by the syphilitic organism—exerts its influence in two places, the fibres ascending to the cord and the postero-median columns. We now know that these both belong to neurons whose nutritive centres are the ganglion-cells of the posterior roots, and we are enabled to infer that the poison has a specific effect upon these neurons. In paralysis agitans, again, we have a ready explanation of the absence of secondary degeneration in the pyramidal tract, for the disease is due to senile decay affecting the dendrons of the cortical cells, but not their axons. As an example of the harmony between processes affecting neuronic terminations at the centre and the periphery we have the similarity between lateral sclerosis and degenerative peripheral neuritis. In each case we have a degeneration beginning in the parts of the axons farthest removed from the nutritive centres; in the first instance this is at the terminations of the pyramidal fibres in the central grey matter of the cord, in the second, at the terminations of the peripheral axons; each may extend a certain distance up the implicated fibres. Even where the application of the new views appears most difficult, as in epilepsy, it may be shown that the conception of such a process starting in nerve-terminations is not more inexplicable than its commencement in the molecules of which the nerve cells are built up. Thus the new facts are of the greatest practical value in aiding the composition of that visual picture of pathological processes which is the sole certain guide to the correct diagnosis and treatment of disease.

